# Phytoplasma infection induces changes in vibrational signals of *Cacopsylla pyri*: sex-specific shifts in frequency, amplitude, and timing

**DOI:** 10.1186/s40850-026-00270-6

**Published:** 2026-05-30

**Authors:** Christina Koller, Ciara Hellweger, Andreas Jürgens, Jürgen Gross

**Affiliations:** 1 Chemical Plant Ecology, Technical University of Darmstadt, Darmstadt, Germany; 2 Julius Kühn-Institute, Federal Research Centre for Cultivated Plants, Institute for Plant Protection in Fruit Crops and Viticulture, Dossenheim, Germany; 3 Department of Crop Protection, Hochschule Geisenheim University, Geisenheim, Germany

**Keywords:** Biotremology, *Cacopsylla pyri*, Mating signals, Phytoplasma

## Abstract

The pear psyllid (*Cacopsylla pyri*), the main vector of ‘*Candidatus* Phytoplasma pyri’, relies on substrate-borne vibrational signals for mate recognition and duet coordination. As pathogens can alter insect communication, we examined whether phytoplasma infection alters key parameters of male and female mating signals. We used laser vibrometry on a standardised artificial substrate, as well as playbacks, to examine this. We detected sex-specific differences in individual signal parameters. In males, infection significantly reduced the amplitude of chirps, whereas uninfected males showed a markedly higher number of interruptions within their trill units. Infected females produced longer inter-chirp intervals and showed higher dominant spectral components than uninfected females. However, fundamental frequency remained stable across treatments, suggesting that differences in dominant frequency reflect shifts in spectral energy among harmonics rather than changes in the primary oscillation frequency. These infection-induced changes may reflect physiological constraints affecting vibrational mechanics. These findings suggest that infection-related differences in dominant frequency reflect changes in the distribution of spectral energy among harmonics rather than a change in the fundamental frequency. Despite these differences at the individual level, the preliminary results of duet communication between males and females did not differ significantly across the various infection treatments. This suggests that the basic coordination in the duet may not be significantly impaired under laboratory conditions; this should be investigated more closely in a specially designed experiment.

**Supplementary Information:**

The online version contains supplementary material available at 10.1186/s40850-026-00270-6.

## Introduction

Pear decline is a disease affecting pear orchards in Europe, causing severe yield losses and long-term economic damage [26]. Symptoms of the disease manifest as a suite of characteristics: infected trees leaf out more slowly in spring, and by late summer or autumn the leaves thicken, curl downwards, and change colour prematurely, often turning red or purple. Fruit size and abundance decrease, while shoot growth is stunted or stops completely [41, 45]. Pear decline occurs in a fast and a slow form with very similar symptoms but a different rate of progression. In Europe, the slow decline is widespread, occurs over multiple seasons and is characterised by a gradual loss of vigour, sparse foliage, and poor yields [44]. The disease is caused by infection of ‘*Candidatus* Phytoplasma pyri’ (‘*Ca.* P. pyri’), a member of the 16SrX group of phytoplasmas [45]. Phytoplasmas are bacteria without cell walls, that rely on phloem-feeding insects for transmission [24, 48]. In insect vectors, it passes through the gut wall, multiplies in the haemolymph, and reaches the salivary glands. From there it can be inoculated into new hosts [10]. This dual life cycle in plants and insects means that vector biology is central to the epidemiology of pear decline.

The primary vector of ‘*Ca*. P. pyri’ in European orchards is the pear psyllid *Cacopsylla pyri* [6]. *Cacopsylla pyri* can be found in orchards throughout the year, in either winter or summer form [22]. Winter-form adults that shelter in bark crevices, whereas summer-form adults are more mobile and consist of up to five or six generations [46]. Early instars nymphs wander to tender shoots to feed. They produce honeydew droplets. Honeydew promotes the growth of sooty mould and can cause russeting of the fruit. High levels of infestation causes ‘psylla shock’, which is a toxin-induced defoliation that diminishes tree vigour [5]. As the adults can survive the winter and acquire phytoplasmas, they play a particularly important role in initiating spring infections. Thus, the life cycle of *C. pyri* determines both the seasonal dynamics and the overall impact of pear decline (Riedle-Bauer et al., [41]).

In recent decades, various alternative control strategies have been developed for the pear psyllid and for other *Cacopsylla* species that act as phytoplasma vectors. Approaches in chemical ecology, biocontrol, and behavioural interventions were developed to disrupt the pest’s life cycle and reduce its impact on fruit orchards. Mayer et al., [34] provided insights into phytoplasma-induced changes in plant volatile emissions and their effect on psyllid attraction and multitrophic interactions. Czarnobai De Jorge et al., [11] investigated a push-and-pull strategy involving pear volatiles, cedarwood and cinnamon bark essential oils [17]. investigated the influence of changes in VOC release over time on the host selection behaviour of *C. pruni* females in olfactory experiments. *Pandora* sp. was evaluated as a biocontrol agent for psyllid control. In an “Attract- and Kill” strategy, this entomopathogenic fungus was used as a “kill component” in combination with specific attractant volatiles [20]. These approaches demonstrate a shift towards behaviour-based interventions in psyllid management; this involves manipulating pest behaviour.

Another promising avenue is mating disruption via biotremology. This approach targets vibrational communication in order to prevent successful mating and offers the potential to be incorporated into an Integrated Pest Management (IPM) strategy. This approach is already well-established in viticulture, where e.g. minishakers are used to disrupt leafhopper communication and consequently reduce population growth (Eriksson et al., 2014). If adult psyllids are prevented from finding mates, their population density is reduced, and consequently the transmission of ‘*Ca.* P. pyri’ is limited. Such an approach would be particularly beneficial for protecting new orchards or healthy fields adjacent to infected sites [27, 31].

Like other psyllids, mating communication in *C. pyri* relies on substrate-borne vibrational signals [1]. Communication is organised as a male-female duet in which males produce calls and actively search for females, whereas females typically remain stationary and respond with chirps when receptive [1, 13, 33]. Male calls usually consist of chirps that may be followed by a trill, while female signals consist of chirps only. During duets, males alternate between walking and signalling, and multiple call-response exchanges can occur. These signal types differ in their temporal organisation and spectral characteristics, including differences in frequency composition.

There are several theories about how psyllids generate vibrational signals. Avosani et al., [1] and Liao et al., [32], for example, assumed stridulation, whereby sound is produced by rubbing body parts together. However, a more recent study by Polajnar et al., [37] compellingly demonstrates that wing buzzing, or rapid wing vibration, is an even more likely mechanism.

To our knowledge, no studies have investigated the influence of a pathogen infection on vibration signals in psyllid species, although it is generally recognised that infections can alter vibrational or acoustic signals in insects. For instance, females of the species *Gryllus lineaticeps*, parasitised by the tachinid fly *Ormia ochracea* respond to a broader range of male signals. This is consistent with an investment strategy under reduced lifespan [40]. Other studies have shown that infections can modify calling signals.

For example, *G. lineaticeps* males infested with *O. ochracea* sang significantly less often than uninfested males, and when they did sing, they sang less frequently [2]. Jacot et al., [25] manipulated the nutritional status of male field crickets *Gryllus campestris* by inoculating them with bacterial lipopolysaccharides. The immune response led to a sustained reduction in calling rate. In psyllids, vibrational communication during mate searching and pair formation is characterised by reciprocal duets in which female replies follow male calls with species-specific latencies and temporal patterns [32]. Therefore, if infection with ‘*Ca*. P. pyri’ alters the temporal or structural properties of male and/or female *C. pyri* calls, this could also reduce the effectiveness of duet initiation and thus reduce mating success.

To characterise these potential infection-induced changes, we focused on key structural and temporal parameters of psyllid vibrational signals; the frequency and the amplitude. Especially in insect communication, a high signal amplitude is crucial for attracting mates or for standing out in a complex acoustic environment [8].

Other parameters, such as the temporal pause between male and female signals, as well as the duration of a complete male call, provide more precise insights into the rhythm and structure of communication in infected and uninfected individuals. These parameters are crucial for understanding how males use temporal intervals in their signals to attract the attention of females [27].

Based on this, we hypothesised that infection with ‘*Ca*. P. pyri’ alters the vibrational communication of *C. pyri*. Specifically, we predicted that the calling signals of infected males and females would differ from those of uninfected individuals in terms of key structural properties (e.g. dominant and fundamental frequency) and temporal (e.g. inter-chirp intervals, call duration) properties. Furthermore, we expected infection to affect female responsiveness during duets, potentially modifying duet formation and thus the dynamics of mate communication.

## Materials and methods

### Insect rearing

*Cacopsylla pyri* individuals were collected using the beating tray method. Species identity was confirmed morphologically included the characteristic forewing pigmentation pattern, male paramere morphology, female terminalia shape, and the structure of the cones. They were kept in net cages (BugDorm-4 Insect Rearing cage, 47.5 × 47.5 × 93 cm) on pear trees (*Pyrus communis* cv. Williams Christ on Kirchensaller rootstocks) in a climate chamber at temperatures ranging from 20 °C to 23 °C and humidity levels ranging from 58% to 62%. A day-night cycle was established, with the lamps on from 6 am to 10 pm. Offspring from the field animals were used for the experiments; these were only summer forms. Young hatched adult males and females were collected twice a week from the cages and were separated by sex to prevent mating to increase the likelihood of being interested in mating during the experiment. They were kept in the cages on ‘*Ca.* P. pyri’ uninfected trees, and then on infected trees via grafting for three weeks before testing, to increase the likelihood of becoming infected.

### Terminology

Vibrational communication in psyllids is typically organised as a male-female duet consisting of a male call and a female reply [32]. The term “signal” refers to the complete vibrational emission produced by an individual. In this study a “call” is defined as a distinct functional signal produced by a male, because in *Cacopsylla pyri* the males call and the females reply. Two call types were observed: (type i) chirp-only calls and (type ii) calls consisting of chirps followed by a trill. A “chirp” is defined as a discrete signal element with a characteristic temporal and spectral structure. Female signals consisted of discrete chirps that could occur either in isolation or in short sequences. A “trill” consists of a sequence of rapidly repeated elements forming a continuous signal segment. The temporal interval between two consecutive chirps is referred to as the inter-chirp interval (pause between chirps). Signal amplitude describes the magnitude of the vibrational signal, and peak amplitude refers to its maximum value. Dominant frequency is defined as the frequency component with the highest energy in the signal spectrum, and fundamental frequency as the lowest frequency component.

### Hardware and software

Mating signals of *C. pyri* were recorded using a laser vibrometer (VibroGo, Polytec GmbH, Waldbronn, Germany). For playback, a linear resonant actuator (LRA; DC-Vibrationsmotor, 3 V, 85 mA, 13500 rpm, Reichelt Elektronik GmbH, Sande, Germany) was connected to an external audio interface (US-2 × 2, Tascam, TEAC Corporation, Tokyo, Japan). Standard audio cables and connectors (e.g. Quarter Inch Stereo Jacks) were used to link the devices.

Recordings were acquired using VibSoft (Polytec GmbH, Waldbronn, Germany) and spectrograms were generated in Raven Pro 1.6 (Cornell Lab of Ornithology, Ithaca, NY, USA). This generated a.wsp file, which was subsequently converted to a.wav file. The playback signals were played using Audacity (open-source software developed by the Audacity Team, version 3.3.3). Vibrational signals were manually segmented and annotated using Raven Pro 1.6 with FFT size of 2048, Hann window, and 50% overlap. Amplitude measurements were obtained from the Raven Pro software and are reported in kilo Units (kU), which are software-specific arbitrary units. These values are not calibrated to physical sound pressure levels and are therefore only suitable for relative comparisons within this study.

To correct for frequency-dependent distortions in the playback devices and to normalise playback amplitudes, the open-source software VibePy [15] was used. VibePy enabled the calibration of the amplitude and correction of device-specific filter effects, thereby ensuring precise and consistent signal transmission. As the same devices (including the LRA) and membrane were used for each measurement, the playback signals only required one-time correction.

### Experimental set up

Experiments were conducted on Petri dishes (9 cm diameter) covered with white printer paper, on which a crosshair-like grid was printed. Nine concentric rings, spaced at 0.5 cm intervals, provided a reference system to normalise the distance of each psyllid from the measurement point. The strength of the vibration signal decreases with distance [23, 35]. To be able to take this effect into account statistically later on, the exact grid position of each psyllid was noted at the time the signal was emitted.

**Fig. 1:**
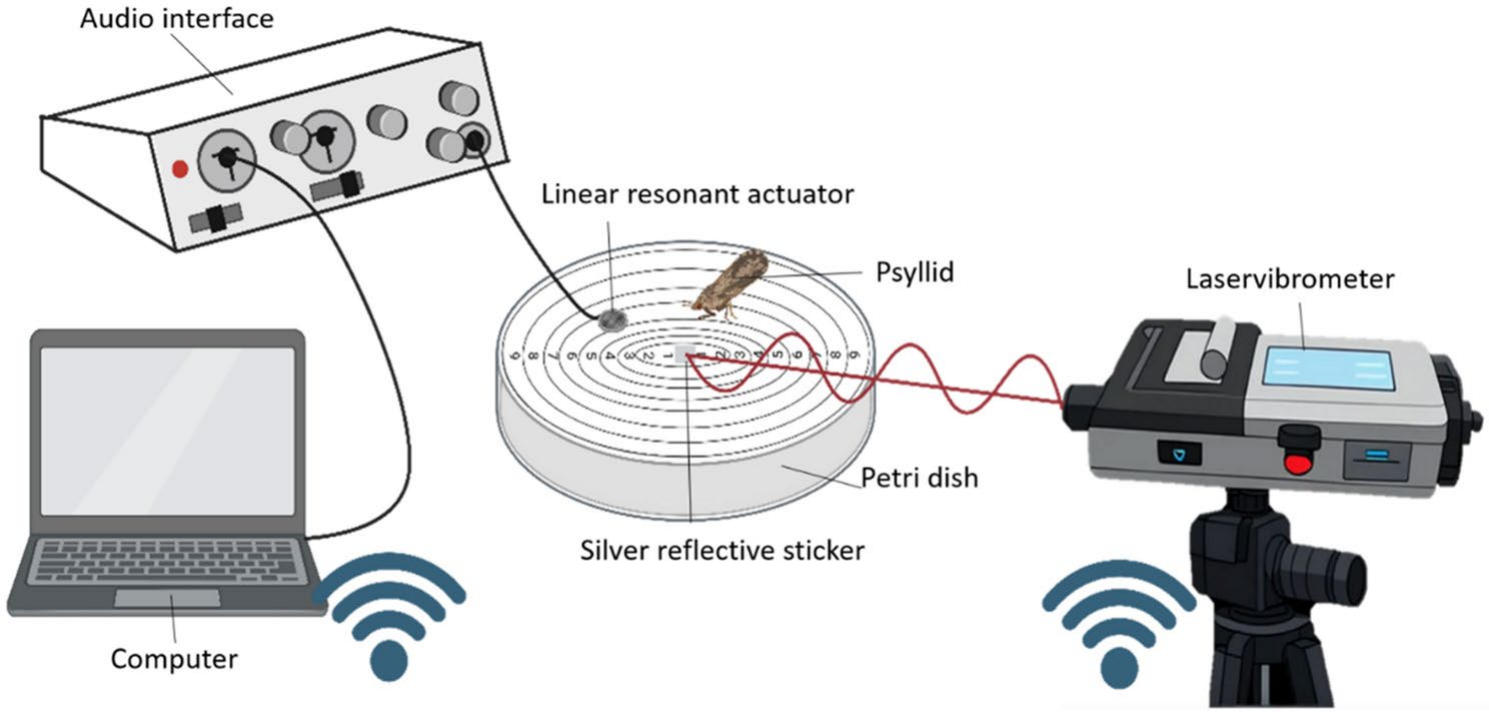
Schematic representation of the setup. The psyllid on the membrane generates vibrations. The laser vibrometer is connected to a computer via Wi-Fi and measures these vibrations. A piece of paper serves as a standardised membrane. Unlike a leaf, its physical properties remain unchanged here, optimising the comparability of the signals. Silver stickers offer the best and most consistent reflective properties for the laser. A linear resonance actuator converts the playback into a vibration signal to stimulate the psyllid. The audio interface reproduces the playback with the same characteristics each time, excluding any influence on the playback from the laptop. Figure created by Christina Koller. Created in BioRender. Gross, J. (2026) https://BioRender.com/a92x829

A silver reflective sticker was applied to the centre of the grid to provide optimal reflection for the laser vibrometer. Vibrations were recorded at a sampling rate if 44.1 kHz. This setup was placed in a Plexiglas box measuring 32 × 32 × 51 cm on top of an anti-vibration balance table (Adam Equipment, United Kingdom). The laser vibrometer was connected to the laptop via Wi-Fi. This setup enabled the precise measurement of both the playback and the insect response signals under controlled and reproducible conditions (see Fig. 1).

#### Playbacks

In order to investigate how infected and uninfected psyllids respond under standardised conditions, we used playbacks in which all individuals received the same stimulus. The male trigger consisted of a sequence of chirps and a trill (total duration: 10.16 s), and the female trigger consisted of four chirps (total duration: 3.3 s). Playback stimuli were presented repeatedly at ~ 5 s intervals until the individual responded or the recording was terminated. Playback amplitude was quantified on the recording dish with a laser vibrometer. Values are reported as peak velocity (mm s⁻¹). Mean peak velocity was 0.14 ± 0.01 mm s⁻¹ for the male trigger (measured over five chirps and the trill) and 0.11 ± 0.02 mm s⁻¹ for the female trigger (measured over four chirps).

The LRA was always positioned in the same place and used consistently. The same male recording was used to trigger females, and the same female recording was used to trigger males. Playback signals were delivered from a laptop via an external audio interface calibrated using VibePy and transmitted to the LRA. Psyllid responses were recorded using the laser vibrometer. Ten infected and ten uninfected males as well as females were tested. The individuals were tested separately and had 10 min to adapt to the environment after being released onto the setup. No individual started signalling without a playback trigger, either in the adaptation or experimental phase.

#### Duets

To study real behaviour in social interactions (“duets”), one male and one female were released onto the set-up together. Playback was only used to stimulate the initial call. Once a real psyllid responded to the playback, no further playbacks were used so that, from then on, only real males communicated with real females. Laser measurement only began after the playback. As the initial response to the playback was sometimes very quick and the laser did not start quickly enough, the initial real signal was excluded from the evaluation in such cases.

Six pairs consisting of uninfected females and uninfected males, four pairs consisting of infected males and females, and four pairs consisting of uninfected females and infected females were tested. The individuals had 10 min to adapt to the environment after being released onto the setup.

### Determining infection status

After recording the signals, each individual was frozen in an Eppendorf tube until extraction and treated as a separate sample. Each individual was mixed with the extraction buffer. The buffer was prepared by mixing CTAB, NaCl, Tris-EDTA, polyvinylpyrrolidone (PVP), and Milli-Q water, then it was autoclaved. Mercaptoethanol was added and then the solution was heated before being mixed and homogenised with the samples. After incubation, chloroform-isoamyl alcohol was added, and the samples were centrifuged. The upper phase was transferred to isopropanol and stored in the freezer for DNA precipitation. The samples were then centrifuged again, and the supernatant was discarded. Ethanol was added, followed by another centrifugation step, after which the supernatant was discarded. Tris-EDTA was then added to resuspend the DNA pellet.

For qPCR, two master mixes were prepared: one to detect psyllid DNA (extraction control) and one to detect phytoplasma DNA (infection control) [4]. Each master mix contained specific primers for the respective DNA targets. Samples or standards were added to a microtiter plate along with controls. The plate was sealed, centrifuged, and then placed in a thermal cycler for qPCR. The procedure is described in detail in Appendix A1.

### Statistics

All statistical analyses were conducted using RStudio (R version 4.4.0) [39]. Figures were generated using the *ggplot2* package [49].

In addition to dominant frequency, we extracted the fundamental frequency (defined as the lowest harmonic component in the signal spectrum) following the recommendation of Polajnar et al., [37]. Fundamental frequency was determined from power spectra (Hann window, FFT size 2048) by identifying the lowest spectral peak within the 0–500 Hz range. Fundamental frequency was analysed at the individual level because it was extracted as one averaged value per individual. Therefore, it was not included in the repeated-measures GLMM framework used for chirp-level parameters, but was compared between infection treatments using Wilcoxon rank-sum tests.

Model fitting relied primarily on generalised linear mixed models (GLMMs), which were implemented in *glmmTMB*. Model assumptions were examined graphically, in accordance with the approach outlined by Zuur et al., [52]. Residual diagnostics were performed using the *DHARMa* package [21], with 1000 simulated residuals. Pairwise contrasts were obtained using *emmeans* on the response scale and adjusted for multiple comparisons with the Holm. Descriptive statistics (means, standard deviations, and sample sizes per treatment) were calculated for all analyses.

Vibrational parameters (dominant frequency and amplitude of chirps and trills, signal duration, male calls (i and ii), and inter-chirp intervals) and female response latency were analysed using gamma GLMMs with a log link. In addition, fundamental frequency was determined from a subset of chirps per individual to assess whether variation in dominant frequency reflected changes in harmonic structure. Individual identity was included as a random intercept to account for repeated measurements. Zero latencies were shifted by + 0.001 s to enable gamma modelling.

Because signal transmission can be affected by the spatial relationship between emitter and recording position, Region was included as a fixed effect. Heterogeneity in residual variance across regions was accounted for using a region-specific dispersion model (dispformula). Gamma GLMMs with a log link and individual identity as a random intercept were used for inference. Estimated marginal means (EMMs) were calculated on the response scale. Due to unequal sampling across regions and individuals, inference was based on model estimates rather than simple descriptive statistics.

The number of interruptions per male call unit (brief breaks within an otherwise continuous trill; count data with overdispersion) was analysed using a negative binomial GLMM. Infection status (infected vs. uninfected) was included as a fixed effect, and male ID as a random intercept to account for repeated call units per individual.

Due to the limited number of duet trials, a descriptive summary is presented in the Results. Inferential analyses were conducted exploratorily and are reported in Appendix A3 only. Female response probability was analysed using a binomial GLMM with female ID as a random intercept. The number of female reply chirps was analysed using a negative binomial GLMM, supplemented by bootstrap-based confidence intervals at the individual level. Response latency was analysed using a GLM with Holm correction for multiple comparisons.

## Results

### Females

**Fig. 2:**
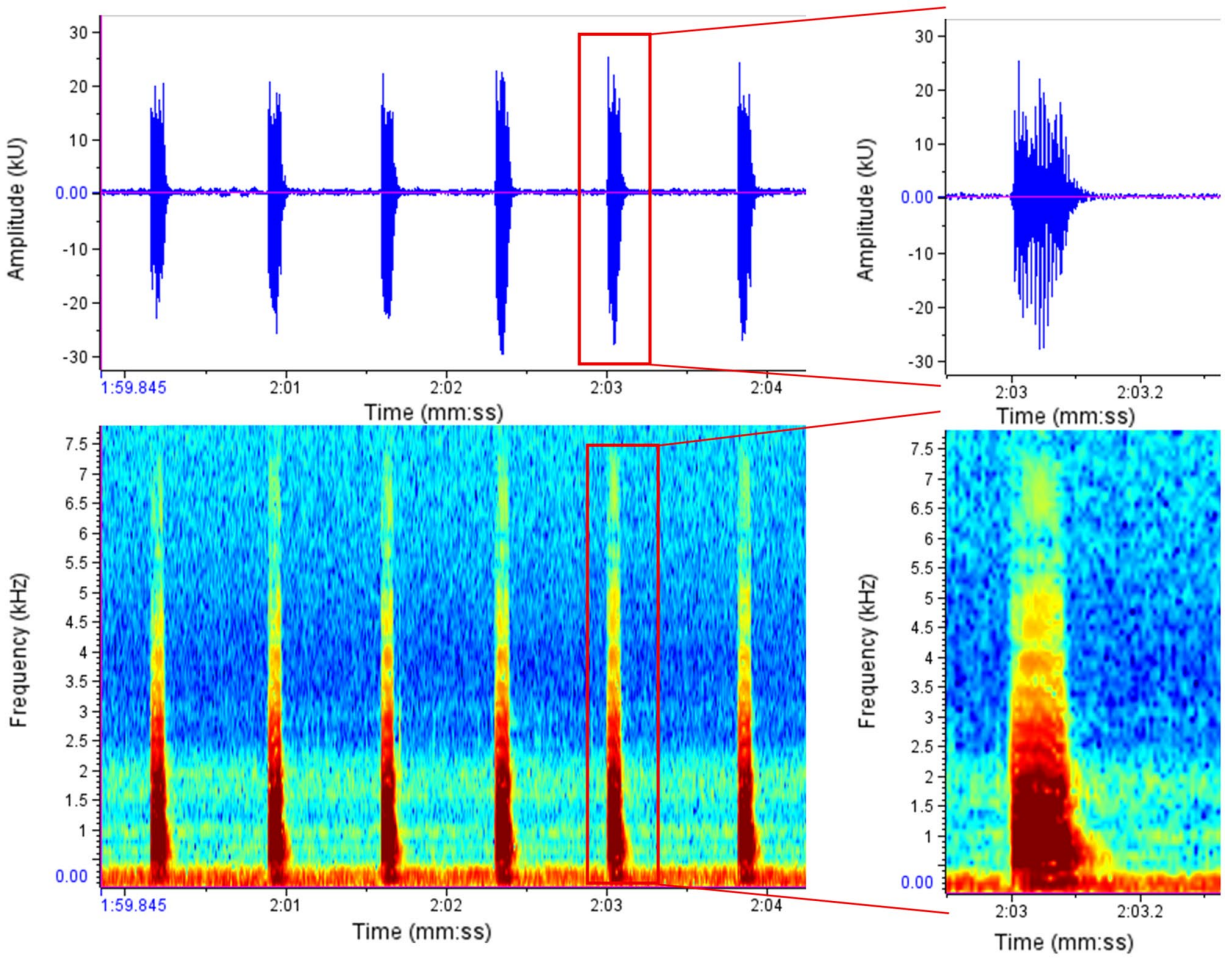
Example of an uninfected female response consisting of several chirps. Peak amplitude is shown in arbitrary units (kU)

Female signals consisted of an isolated chirp or several chirps in short sequences. In most female signals, the chirps occurred one after the other, with a clear separation between them, so that they never appeared directly next to each other (Fig. 2).

**Fig. 3:**
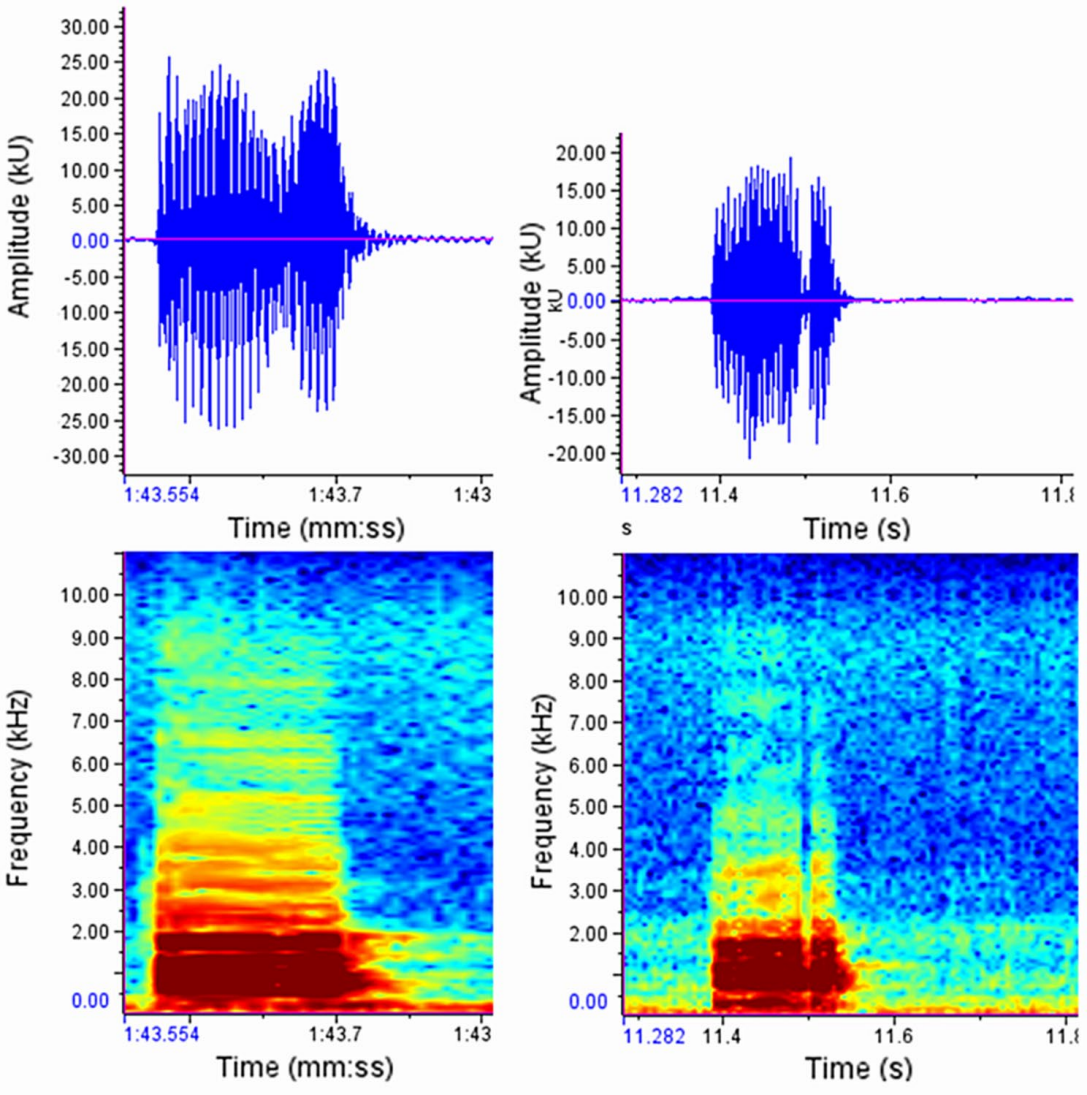
Examples of female signals. The left panel shows a double chirp consisting of two temporally separated components. The right panel shows a signal that acoustically resembles a double chirp but appears as a single chirp with internal frequency modulation in the spectrogram. Classification was based primarily on oscillogram structure and auditory inspection. Due to the FFT size (2048), closely spaced components may not be fully resolved in the spectrogram

In addition, some of the female chirps appeared as ‘double chirps’ defined as elements consisting of two closely spaced components separated by a brief reduction in amplitude in the oscillogram (Fig. 3). In some cases, signals that acoustically resembled double chirps showed a continuous structure in the spectrogram, consistent with internal frequency modulation rather than two distinct signal events. Due to the FFT size used (2048), closely spaced components were not always fully resolved in the spectrogram, and classification was therefore based primarily on oscillogram structure. These double chirps were described for the first time in our study and occurred less frequently than the single chirps, which have already been described [13].

One individual from each treatment group was excluded from this analysis because the recordings did not allow reliable identification of double chirps due to signal quality. The final sample therefore comprised nine uninfected and nine infected females. Seven out of nine females in each treatment exhibited double chirps. For each of the nine individuals per treatment, 50 chirps were examined and classified as single or double. Uninfected individuals exhibited a median of 9 (mean 8.4, SD = 6.06), while infected individuals exhibited a median of 7 (mean 8.0, SD = 6.96) double chirps. This difference was not significant (Wilcoxon rank-sum test; W = 42, *p* = 0.929).

**Fig. 4:**
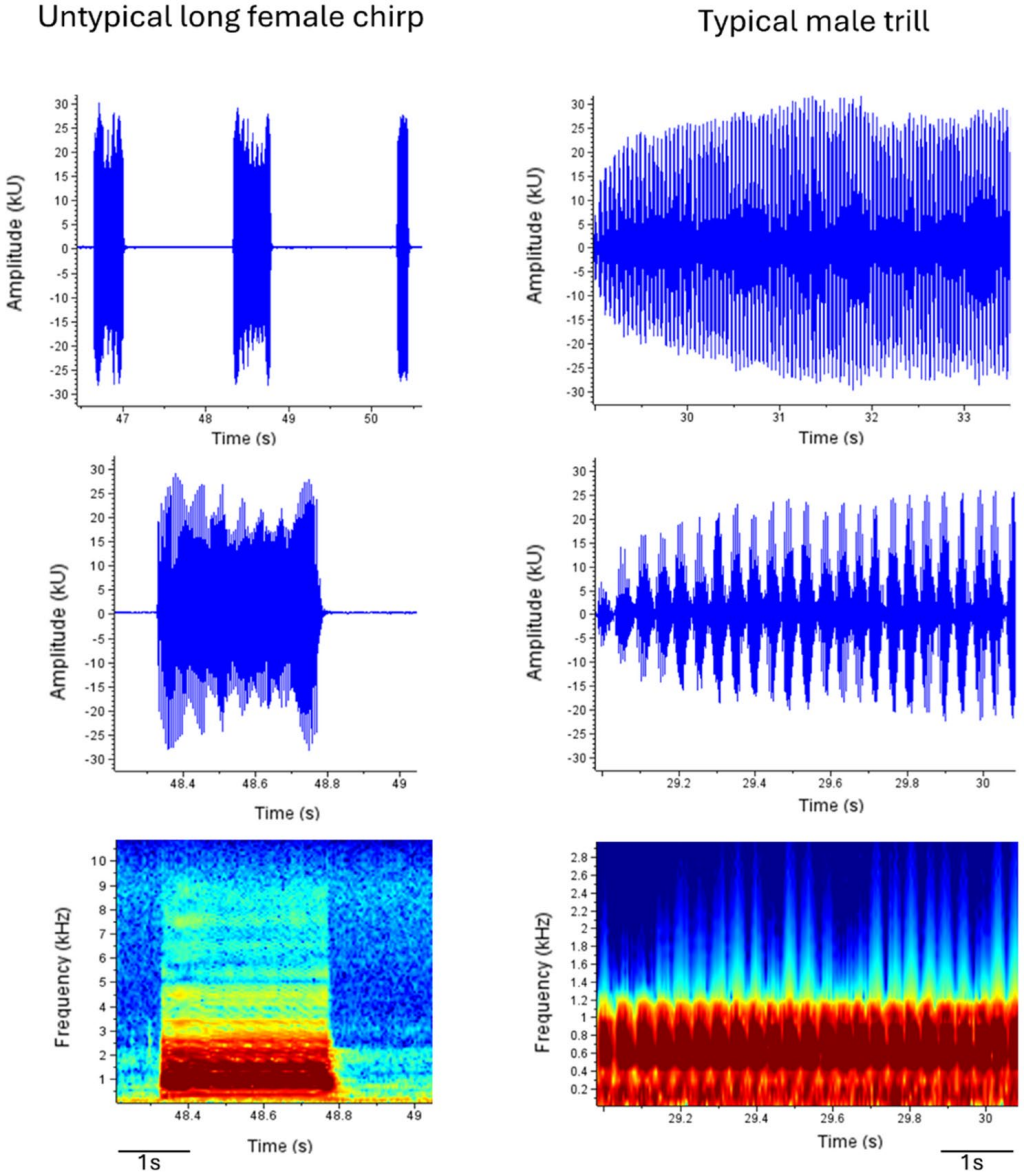
Comparison of an unusually long female chirp (infected) and a typical male trill. The female signal forms a continuous event, whereas the male trill consists of rapidly repeated discrete elements

In one infected female, we observed an unusually long chirp that acoustically resembled a male trill but differed in its temporal structure (Fig. 4). Unlike male trills, which consist of rapidly repeated discrete elements, the female signal formed a single continuous event in the oscillogram. This phenomenon was repeatedly observed within this individual (36 out of 91 chirps), but was not detected in other individuals. Given that this observation was limited to a single individual, it is presented here as a descriptive example of signal variation.

Fifteen chirps and 15 intervals between two chirps were randomly selected from each individual. Model-based summary statistics for female signal parameters are provided in Table 1, and descriptive statistics based on raw data are given in Appendix Table 5. We examined the fundamental frequency (lowest harmonic component) to assess whether infection affected the primary oscillation frequency. No significant differences between infected and uninfected individuals were detected (Wilcoxon rank-sum test; females: W = 31.5, *p* = 0.17). Mean fundamental frequency was 201.12 ± 1.81 Hz (0.201 ± 0.002 kHz) in uninfected females and 199.40 ± 2.68 Hz (0.199 ± 0.003 kHz) in infected females, indicating comparable values between treatments.

**Table 1:** Summary of vibrational signal parameters in infected and uninfected female *C. pyri*. Values represent model-based estimated marginal means (EMMs ± SE), adjusted for region and repeated measurements per individual relative to the infected reference level

Parameter	Uninfected EMM ± SE	Infected EMM ± SE	*N* (uninf./inf.)	*n* (uninf./inf.)
Inter-chirp pause (s)	0.643 ± 0.072	0.848 ± 0.147	10	150
Chirp duration (s)	0.141 ± 0.019	0.146 ± 0.033	10	150
Dominant frequency (kHz)	4.535 ± 0.102	5.335 ± 0.204	10	150
Peak amplitude (kU)	1.878 ± 0.223	1.987 ± 0.403	10	150

**Fig. 5:**
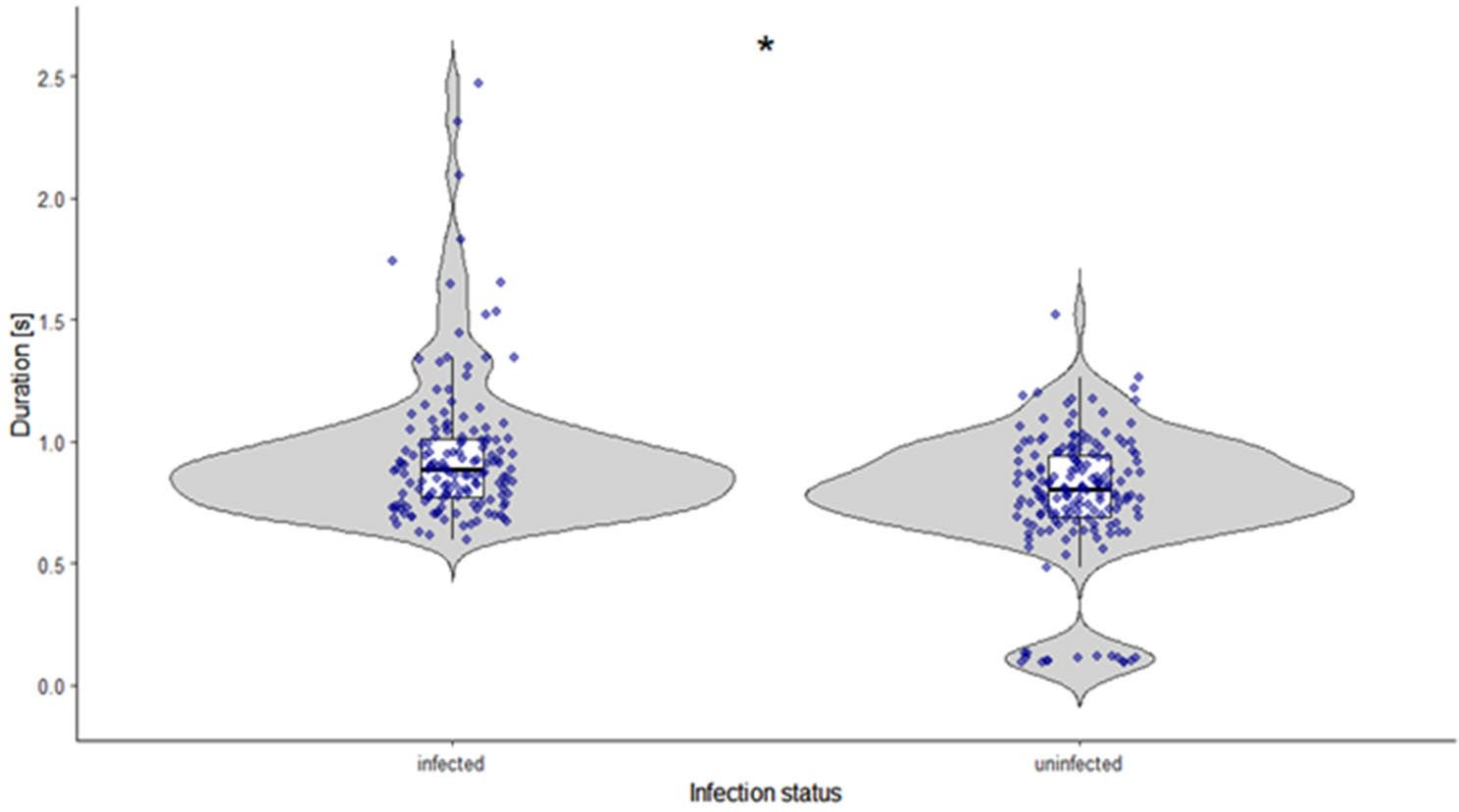
Difference in pause (s) between chirps of ‘*Ca*. P. pyri’ infected (*N* = 10, *n* = 150) and uninfected (*N* = 10, *n* = 150) *C. pyri* females. Points represent individual chirps (raw data); statistical inference accounts for repeated measurements per individual. Boxplots indicating the median and interquartile range

**Fig. 6:**
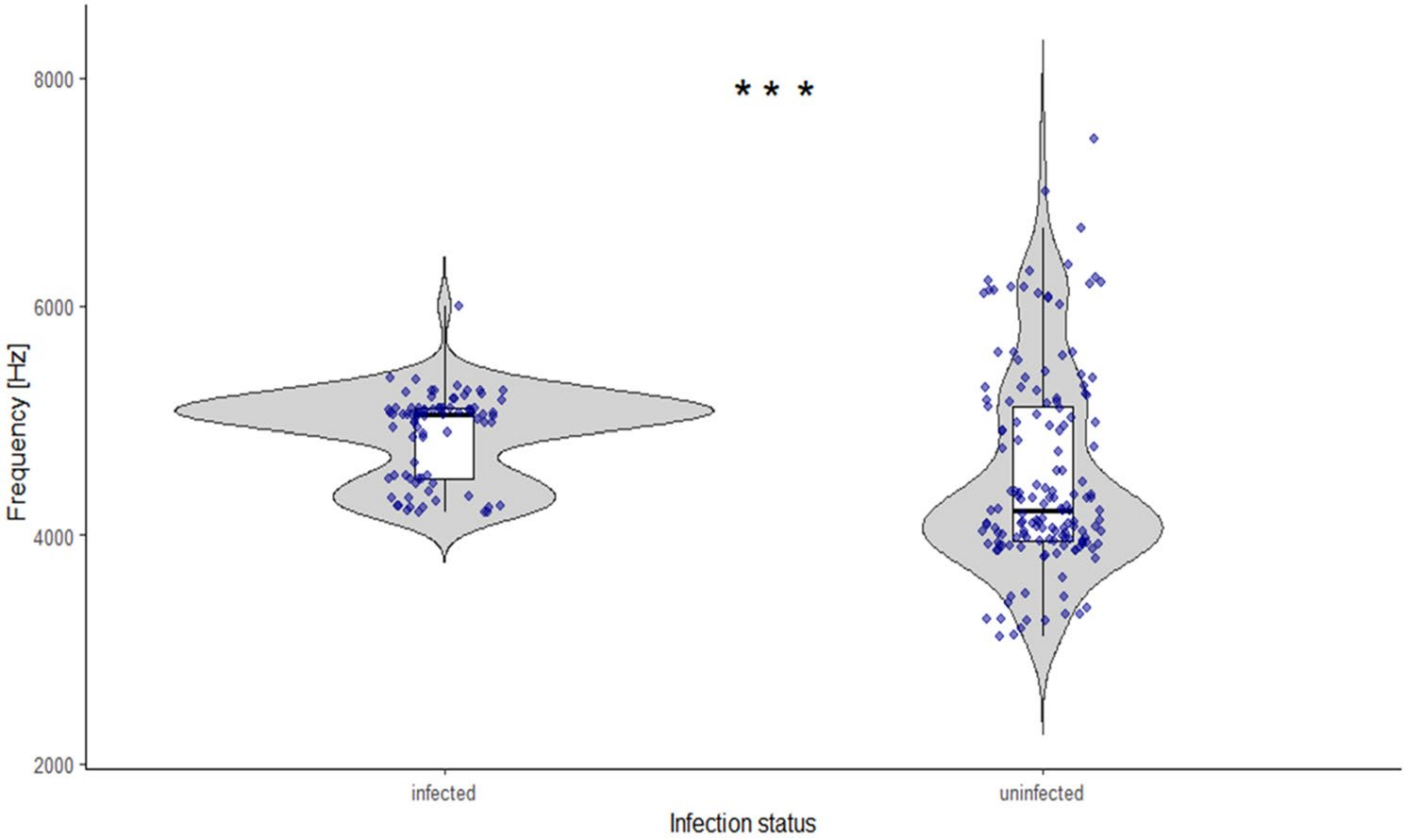
Difference in dominant frequency (kHz) between chirps of ‘*Ca*. P. pyri’-infected (*N* = 10, *n* = 150) and uninfected (*N* = 10, *n* = 150) *C. pyri* females.Points represent individual chirps (raw data); statistical inference accounts for repeated measurements per individual. Boxplots indicating the median and interquartile range

No significant effects of infection were detected for chirp duration or peak amplitude in female *C. pyri* (Gamma GLMMs, all *p* > 0.05; Table 4 in Appendix A2). However, infection had a significant effect on both inter-chirp pause (Gamma GLMM, *p* = 0.012; Table 4 in Appendix A2) and dominant frequency of chirps (Gamma GLMM, *p* = 0.00014; Table 4 in Appendix A2). Model-based estimates indicated longer inter-chirp pauses and higher dominant frequency values in infected females than in uninfected females (Table 1; Figs. 5 and 6). In uninfected females, most chirp durations were concentrated within a narrow range, while a small number of shorter chirps resulted in a secondary cluster at lower values. The distribution of dominant frequency in uninfected females showed two distinct clusters.

#### Males

**Fig. 7:**
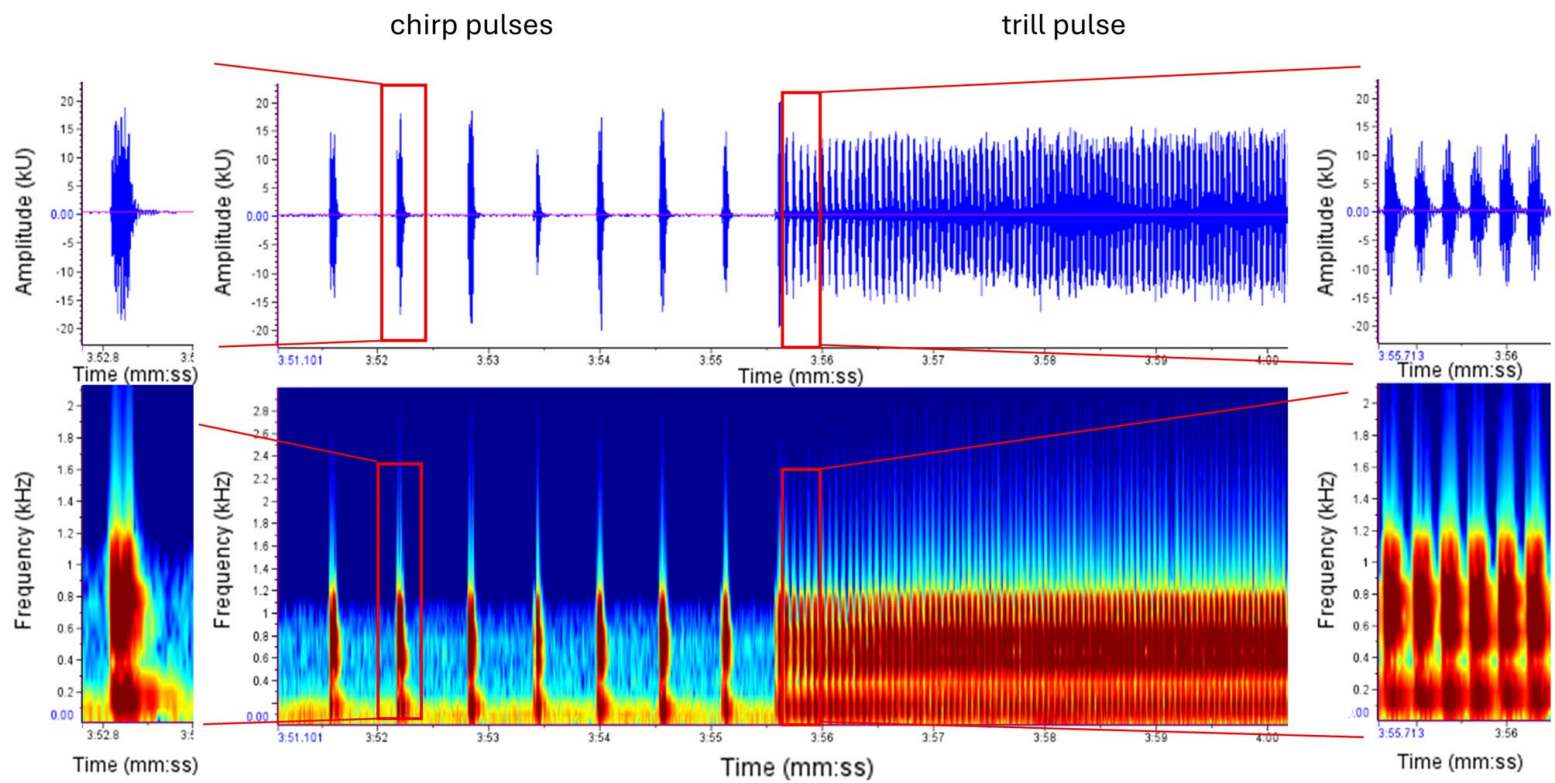
Example of a uninfected male call (type ii): call consists of several chirps and a following trill. In this case, a chirp amplitude of approximately + 18 and − 18 kU was achieved

It is more difficult to obtain the same samples for trills than for chirps because, in general, only one continuous trill follows many chirps. A call (type ii) consists of chirps and the trill (Fig. 7).

From each individual, 15 chirps, 15 intervals between two chirps, 6–20 trills and 3–10 calls (type ii) were randomly selected. Model-based summary statistics for male signal parameters are provided in Table 2. and descriptive statistics based on raw data are given in Appendix Table 5. We examined the fundamental frequency (lowest harmonic component) to assess whether infection affected the primary oscillation frequency. No significant differences between infected and uninfected individuals were detected (Wilcoxon rank-sum test; males: W = 51, *p* = 0.97). Mean fundamental frequency was 195.40 ± 11.39 Hz (0.195 ± 0.011 kHz) in uninfected males and 199.54 ± 1.93 Hz (0.199 ± 0.002 kHz) in infected males, indicating comparable values between treatments.

**Table 2:** Summary of vibrational signal parameters in infected and uninfected male *C. pyri*. Values represent model-based estimated marginal means (EMMs ± SE), adjusted for region and repeated measurements per individual

Parameter	Uninfected EMM ± SE	Infected EMM ± SE	*N* (uninf./inf.)	*n* (uninf./inf.)
Inter-chirp pause (s)	0.624 ± 0.173	0.841 ± 0.234	10	150
Chirp duration (s)	0.075 ± 0.005	0.082 ± 0.005	10	150
Dominant frequency (kHz)	2.513 ± 0.417	2.651 ± 0.440	10	150
Peak amplitude (kU)	1.344 ± 0.153	1.057 ± 0.120	10	150
Call (type ii) duration (s)	22.123 ± 0.938	23.147 ± 1.003	5	27/24
Trill duration (s)	15.378 ± 0.763	15.622 ± 0.688	4/5	21/26
Trill peak amplitude (kU)	2.561 ± 0.344	3.185 ± 0.426	4/5	21/26

**Fig. 8:**
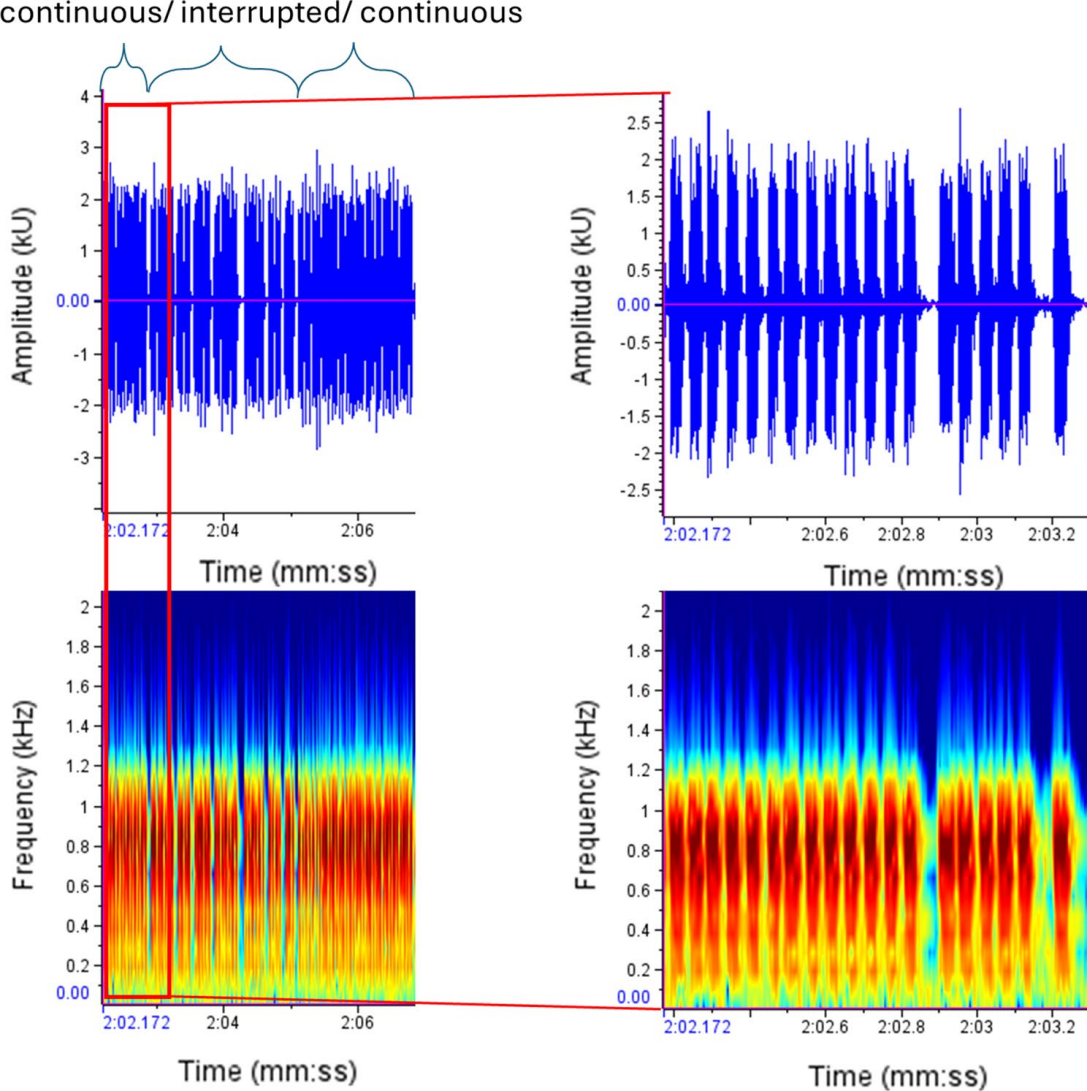
Example of a male trill containing both continuous and interrupted segments

In some individuals, interruptions in the trill could be observed. The chirps are followed by the trill, which then breaks off in several places and continues after less than 1/10 second. This phenomenon has been observed in both infected and uninfected *C. pyri* (Fig. 8).

**Fig. 9:**
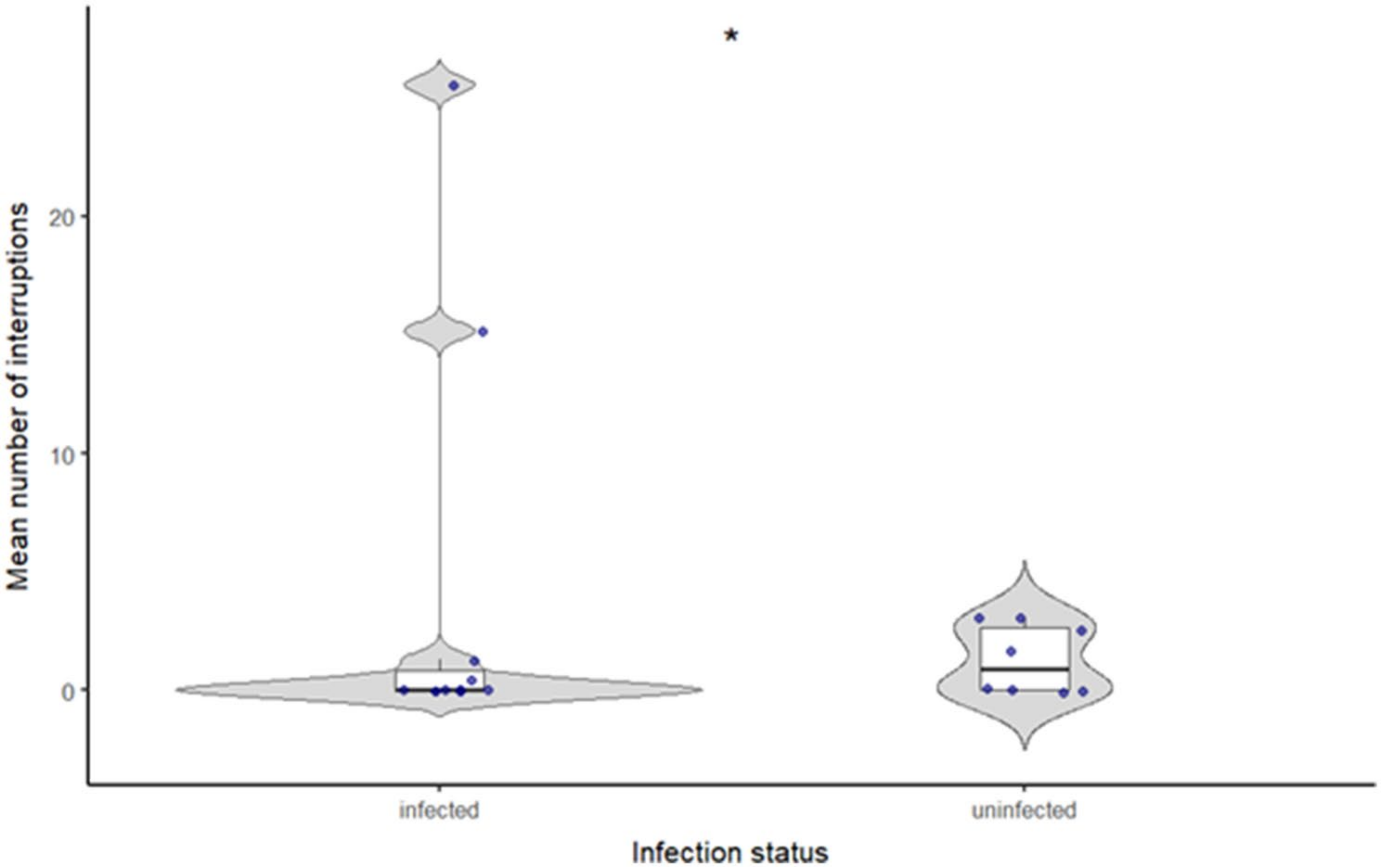
Interruptions within male call units in relation to infection status (uninfected males: *N* = 8, *n* = 56, infected males: *N* = 11, *n* = 106). Points represent individual trills (raw data); statistical inference accounts for repeated measurements per individual. Boxplots indicating the median and interquartile range

Uninfected males (*N* = 8, *n* = 56) produced significantly more interruptions within their trill units than infected males (*N* = 11, *n* = 106) (negative binomial GLMM, *p* = 0.021; Fig. 9). Individual differences contributed substantially to variation, as captured by the random effect of male identity. The unequal number of analysed signals and trills between treatments reflects natural variation in male calling behaviour. Some males produced more chirp-rich calls or repeated trills more frequently, while others reduced trill production over time. Only trills of comparable quality were included, which further affected the final sample size.

**Fig. 10:**
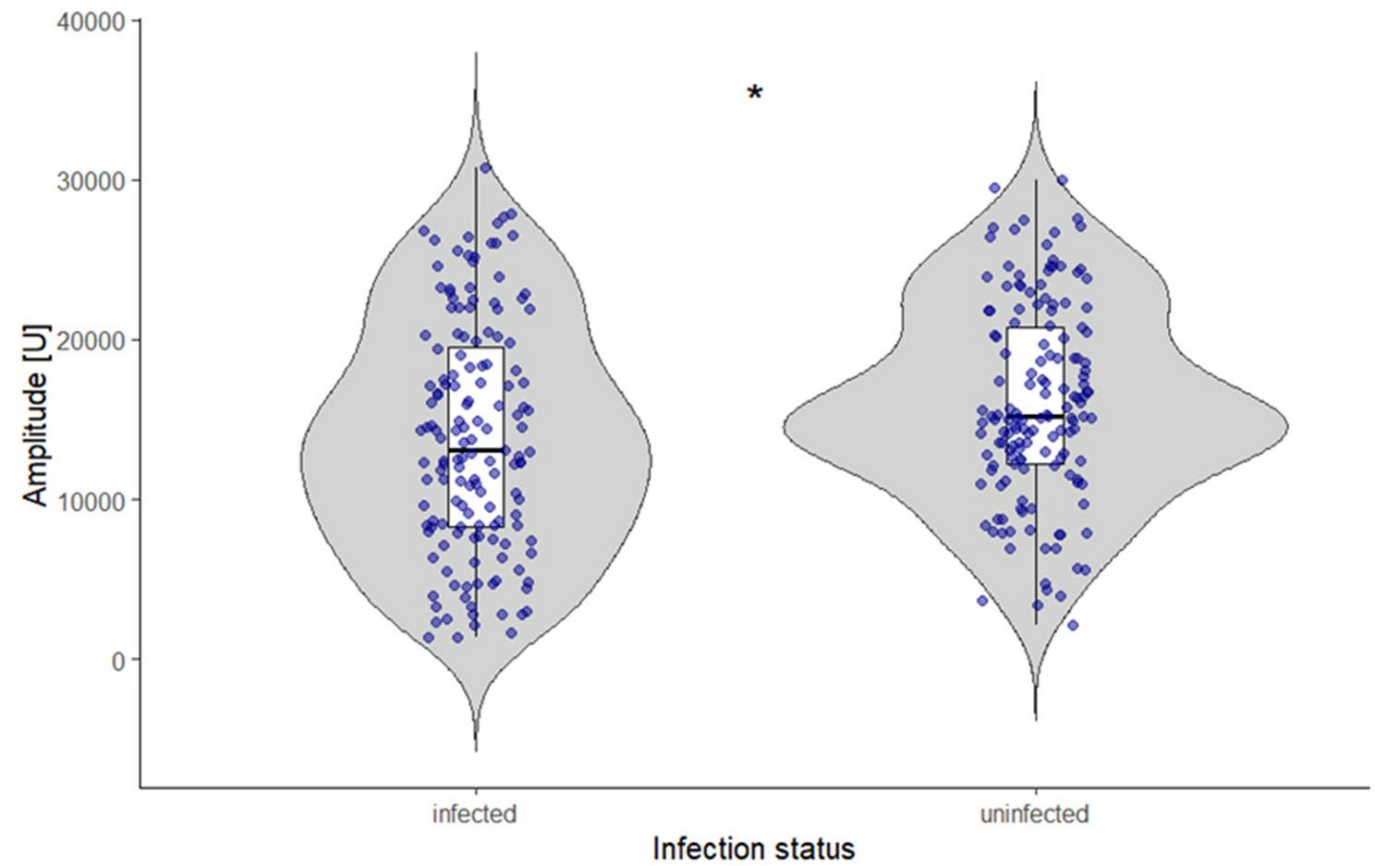
Peak amplitude of male chirps in relation to infection status (uninfected males: *N* = 10, *n* = 150, infected males: *N* = 10, *n* = 150). Points represent individual chirps (raw data); statistical inference accounts for repeated measurements per individual. Boxplots indicate the median and interquartile range. Individual male identity was included as a random effect to account for repeated measurements

A significant effect of infection was detected for male chirp peak amplitude (Gamma GLMM, *p* = 0.039; Table 4 in Appendix A2), with model-based estimates indicating lower peak amplitudes in infected males than in uninfected males (Table 2; Fig. 10). No significant effects of infection were detected for male inter-chirp pause, dominant frequency of chirps or trills, call duration, or trill peak amplitude (Gamma GLMMs, all *p* > 0.05; Table 4 in Appendix A2).

### Duets

**Fig. 11:**
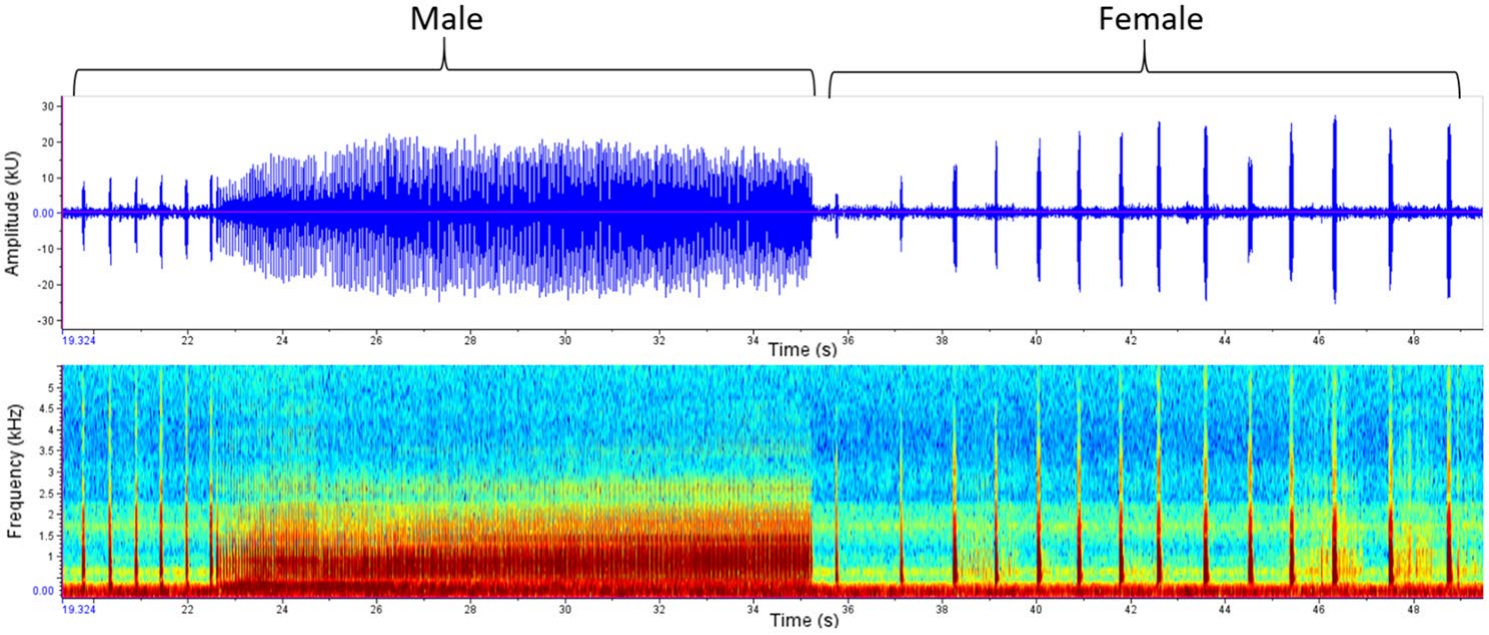
Example of a duet of an uninfected male and an uninfected female. Male calls (type ii) are typically several chirps and a long trill and female responded right after less than 0.5 s with several chirps

Treatments were abbreviated as UU (uninfected female + uninfected male), UI (uninfected female + infected male) and II (infected female + infected male). A total of 14 duets were evaluated. A duet contains a male call and a female response (Fig. 11). A duet between an infected female and an uninfected male could not be recorded. A duet did not necessarily result in copulation. Given the limited number of duet trials and the uneven representation of infection combinations, duet data are presented descriptively in Table 3. A larger sample size was not achievable due to the declining laboratory population. To provide an outlook on a potential analytical strategy for larger datasets, inferential statistical models are presented in Appendix A3; however, these models are not used as a basis for interpretation.

Mating occurred in two UU pairs, one UI pair and one II pair. In two additional UI interactions, females rejected the courting male.

**Table 3:** Descriptive summary of duets across infection combinations in *Cacopsylla pyri*. Treatments are UU: uninfected female × uninfected male; UI: uninfected female × infected male; II: infected female × infected male. Latency refers to the time interval between male call and female response. Chirp number refers to the number of female chirps within a single response

Parameter	Uninfected female vs. uninfected male	Uninfected female vs. infected male	Infected female vs. infected male
Number of duets (N)	6	4	4
Female responses analysed (n)	29	14	15
Median response latency (sec.)	0.39	0.32	0.24
Minimum latency (sec.)	0.00	0.06	0.00
Maximum latency (sec.)	23.84	3.34	1.95
Median female chirps per response	10	8	33
Minimum chirps per response	1	2	3
Maximum chirps per response	68	89	98

## Discussion

### Females

Our results show that ‘*Ca.* P. pyri’ infection is associated with differences in certain vibrational signal parameters in female *C. pyri*. Infected females exhibited significantly longer inter-chirp intervals and higher dominant frequencies, while other parameters, such as chirp duration and amplitude, remained largely unaffected. The energetic cost per chirp bout may therefore be lower in females, potentially explaining why infection did not significantly affect female amplitude.

Longer pauses between chirps could reflect a neuromotor burden associated with infection, whereby energy is redirected towards pathogen replication rather than signalling. Similar energetic trade-offs have been reported in other insect-microbe interactions, where infections or symbionts influence host resource allocation and behaviour [28, 38]. In a vector-pathogen context, Killiny et al., [29] demonstrated that ‘*Candidatus* Liberibacter asiaticus’ alters energy metabolism in its vector *Diaphorina citri*, supporting the idea that pathogen-mediated physiological changes may affect energetically costly behaviours. The broader and partially bimodal distribution of dominant frequency in uninfected females indicates elevated within-group variability, although whether this reflects biological variability or infection-related stabilisation remains unclear.

To interpret these results, it is necessary to distinguish between dominant frequency, which reflects the strongest spectral component, and fundamental frequency, which represents the primary oscillation frequency. Wenninger et al., [50] found that dominant frequencies in *D. citri* signals reflect the frequency range relevant for communication, including both fundamental and dominant components. Similar patterns have been reported in other insect species using vibrational signals for mate attraction and communication [13, 19]. We observed higher dominant frequencies in infected females. However, fundamental frequency did not differ between infection treatments in either sex, indicating that the primary oscillation frequency remained stable. Therefore, the observed differences in dominant frequency are best interpreted as a shift in the dominant spectral component rather than as evidence for an altered signal-production mechanism. Such shifts may reflect differences in harmonic structure and/or transmission-related effects (e.g. position-dependent variation in harmonic structure). The present study design does not allow disentangling effects of signal production from transmission-related changes. In general, signal frequency is more strongly influenced by morphological and structural features than by energy availability [1]. Xing and Yang, [51] demonstrated that the stiffness distribution within insect cuticle strongly influences the mechanical properties of the exoskeleton. One possible explanation may be that infection could alter mechanical properties of the signal-producing system, including exoskeleton stiffness, mass, or coupling to the substrate, which could in turn affect resonance and thereby shift the dominant frequency.

Dominant frequencies may also alter signal transmission on plant substrates, potentially reducing long-range propagation but enhancing detectability over short distances. Eriksson et al., [14] documented similar effects in *Scaphoideus titanus* during substrate-borne vibrational communication. Changes in female signal properties could reduce mating efficiency when males attempt to contact infected partners [47]. If infected females are less successful in exchanging signals, the reproduction of infected individuals may decline. However, if infection does not substantially reduce survival, infected individuals may remain infectious for longer periods. This could increase the relative proportion of infectious, long-lived vectors within the population, as reported in other vector–pathogen systems [43].

The absence of differences in signal amplitude or duration suggests that the capacity for signal production is largely retained. Therefore, infection appears to affect the fine control of rhythmic and spectral patterns rather than overall signal energy.

### Males

The results suggest that the infection is associated with differences in the signalling behaviour of males, particularly in terms of amplitude. Other aspects of the signal, such as trill structure, are largely unaffected. In this species, chirps are short, high-amplitude signal elementsthat require substantial energy to produce, whereas trills are longer but lower in amplitude and therefore less energetically costly. This observed reduction in male chirp amplitude should, however, be interpreted with caution. Although the effective sample size was identical for both sexes, the comparatively modest male amplitude effect occurred in the context of visible inter-individual variability. With a limited number of independent individuals, treatment differences in males may be more sensitive to individual outliers than the corresponding female effects, which were statistically stronger and associated with more homogeneous distributions. Studies have shown that the peak amplitude can indicate the extent to which signal elements (chirps) are used for communication in dense habitats or during intense behavioural interactions [7]; Wenninger et al., 2019). One possible explanation is that the phytoplasma diverts the psyllids’ energy resources for its own reproduction, thereby limiting the energy available for energy-intensive components such as chirps. The redistribution of resources in response to an immune activation has been documented in crickets [16], where males with a stronger immune response exhibited altered mating signals. Importantly, the preservation of trills suggests that the basic attractiveness to mates is maintained, may not necessarily disrupt basic coordination under lab conditions. Gesto et al., [18] further support this idea, having observed alterations in the mating signals of *Wolbachia*-infected male and female *Aedes aegypti* without completely disrupting mating. Although *Wolbachia* represents an endosymbiont rather than a plant pathogen transmitted by insect vectors, these studies illustrate how microbial associations can influence host signalling behaviour. Our findings indicate selective modulation of sexual signals by phytoplasma infection rather than a general decline in performance.

This interpretation is supported by Koller et al., [30], who showed that infection alters energy storage, particularly sugar reserves, in *C. pyri*. In relation to our results, the reduction in chirp amplitude in male signals or the prolonged pauses in female signals could precisely reflect these resource transfers. We suspect that energy may be diverted to replicate the phytoplasma in the host, leaving less available for energetically costly signalling activities. Sex-specific differences in energy storage (e.g. higher lipid content in males) and signalling behaviour (e.g. amplitude in males and frequency in females being differentially affected) may reflect broader infection-related shifts in resource allocation. Comparable reduced vector fitness following phytoplasma exposure has been demonstrated in *Scaphoideus titanus*, where infection was associated with physiological costs [3]. In addition, vector performance has been shown to depend on the context of the host-plant in phytoplasma systems, highlighting the importance of plant-vector-pathogen interactions [42].

### Duets

Within the present descriptive dataset, no clear differences in duet structure across infection combinations were apparent. Given the small number of interacting pairs, these observations should be regarded as preliminary.

We tentatively hypothesise that infection-induced signal modifications do not fundamentally disrupt duet coordination under laboratory conditions. Comparable infection-related alterations without collapse of duet interaction have been reported in other systems (e.g. Wolbachia in Aedes aegypti; [18]), although transmission mode and host-microbe relationships differ substantially.

As the physical properties of natural substrates (particularly the damping, conductivity, and resonance effects of pear leaves) significantly influence on signal transmission [9, 36], the impact of infection on mating in real conditions cannot be inferred directly from our laboratory results. Subtle changes in frequency, amplitude, or timing in a natural leaf environment could affect partner localisation or copulation probability. Future experiments under natural conditions would therefore be necessary to conclusively assess the functional consequences of infection on reproductive success. In addition, in our study, adults were exposed to infected host plants for three weeks prior to behavioural testing. Future work could assess whether earlier infection, for example during juvenile development, results in stronger or qualitatively different effects on vibrational signalling. Developmental exposure may lead to higher pathogen titres or prolonged physiological modulation, potentially amplifying infection-associated behavioural changes.

Accordingly, we suggest that preventing mating through playbacks may act as a context-dependent interference, primarily affecting individuals during active mate searching and duet formation, whereas infection status appears to play no role in this process. For potential applications, we recommend combining playbacks with other approaches, such as coloured sticky traps [12].

## Conclusion

Our study demonstrates that phytoplasma infection alters vibrational communication in *Cacopsylla pyri* in a sex-specific manner. While modifications were detected at the level of individual signal parameters, no consistent differences in duet organisation were observed under the controlled laboratory conditions used here. These findings indicate that infection-related changes affect specific temporal and spectral components of signalling without necessarily disrupting basic duet coordination. However, because mating success and transmission dynamics were not directly assessed, the functional consequences of these modifications remain uncertain.

Future studies under semi-natural or field conditions will be required to determine whether infection-associated signal changes influence reproductive outcome or pathogen spread.

## Supplementary Information

Below is the link to the electronic supplementary material.

Supplementary Material 1

Supplementary Material 2

Supplementary Material 3

Supplementary Material 4

## Data Availability

The data are available from the corresponding author upon reasonable request.

## Abbreviations

CTAB: Cetyltrimethylammonium bromide
EDTA: Ethylenediaminetetraacetic acid
MilliQ: ultrapure water (Milli-Q^®^ system)
NaCl: Sodium chloride
PVP: Polyvinylpyrrolidone
qPCR: Quantitative polymerase chain reaction
RH: relative humidity
TRIS-EDTA: Tris(hydroxymethyl)aminomethane–EDTA buffer
VOC: Volatile organic compound
