# Supplementary material for: Phytoplasma infection induces changes in vibrational signals of Cacopsylla pyri: sex-specific shifts in frequency, amplitude, and timing
Source: BMC Zool. 2026 May 30;11:19. doi: 10.1186/s40850-026-00270-6 (PMC13227762; doi:10.1186/s40850-026-00270-6)
Supplement: Supplementary file 1 — Supplementary Material 1 [file 40850_2026_270_MOESM1_ESM.docx]

A1

DNA-Extraction and qPCR in detail

1 L buffer was prepared, consisting of 25 g CTAB (Cetyl trimethyl ammonium bromid), 81.9 g NaCl, 100 ml TRIS-EDTA pH 8, 40 ml EDTA, 10 g PVP, MilliQ water ad 1L (Doyle, 1991). This buffer was autoclaved. To each 50 µl sample 500 µl buffer with 0.2% mercaptoethanol ((Merck KGaA, Germany) was added. Mercaptoethanol was added fresh to the Doyle buffer for each extraction. The final buffer was heated at 60 °C in a water bath (VWR International GmbH, Germany) for 30 min before being added to the samples. The samples were crushed fpr 1 minute in the fast prep. Then incubated for 30 min at 60 °C on the thermoblock. During this time, 350 µl Isopropanol (Zentrallager Universität Heidelberg, Germany) was added to each 2 ml save-lock reaction vial and then stored in the freezer. After incubation, 500 µl of chloroform-Isoamyl alcohol was added to each sample and then centrifuged for 6 min at 12.700 rpm. Phase formation took place. Approximately 350 µl of the upper phase was removed with a pipette and transferred to the prepared save-lock reaction vials containing isopropanol. The samples were then stored in the freezer for 1h for DNA precipitation.

The samples were then centrifuged at 12.500 rpm and 4 °C for 20 min. The supernatant was tilted and discarded. Subsequently, 300 µl ETOH (70 %) was added and centrifuged at 12500 rpm for 6 min. The supernatant was discarded. Then 50 µl TRIS-EDTA was added to each sample and the DNA pellicle was dissolved by resuspension.

A multiplex approach was prepared for the qPCR to check the sample for the presence of plant DNA (extraction control) and to check the sample for the presence of phytoplasma DNA (infection control). It was assumed that psyllids that had been feeding on the phloem prior to being caught on sticky traps would contain traces of plant material in their gut at the time of extraction. Consequently, if no plant DNA was detected during PCR amplification, the respective DNA extraction was considered unsuccessful and the sample was excluded from further analysis. The amount of master mix components per sample was 8,25 HPLC, 12.5 µl Master Mix (2x primaQUANT, Steinbrenner Laborsysteme GmbH, Wiesenbach, Germany), 0.25 µl fPlant (forward primer 5-GAC TAC GTC CCT GCC CTT TG -3’), 0.25 µl rPlant (reverse primer 5- AAC ACT TCA CCG GAC CAT TCA-3), 0.25 µl Cy5, 1 µl fChrist (forward primer 5-CGTACGCAAGTATGAAACTTAAAGGA-3’), 1 µl rChrist (reverse primer 5-TCTTCGAATTAAACAACATGATCCA-3), 0.5 µl FAM (Christensen, 2004).

For each sample, 24 µl of the master mix was added in a microtitre plate. Then 1 µl each of the previously vortexed sample or 1 µl each of the vortexed standard series (10^-1^, 10^-2^, 10^-3^, 10^-4^, 10^-5^, 10^-6^, 10^-7^ und 10^-8^ DNA-copies/μl), positive and negative control (known infected and non-infected tobacco) was added. The microtitre plate was sealed with a foil (Biozym Scientific GmbH, Deutschland) and centrifuged in the plate centrifuge (BMG Labtech, FluoStar Omega) for 10 sec. qPCR was carried out in a thermocycler (CFX96 Real-Time System C1000 Touch Thermal Cycler, Bio-Rad Laboratories GmbH, Germay). Initial denaturisation took place for 3 minutes at 95°C. Annealing took place at 95 °C for 10 s, and elongation at 65 °C for 30 s. The cycle was repeated 40 times.

References:

Doyle, J. DNA Protocols for Plants. In: Hewitt, GM, Johnston, AWB, Young, JPW, editors. Molecular Techniques in Taxonomy. NATO ASI Series. Volume 57. Berlin, Heidelberg: Springer; https://doi.org/10.1007/978-3-642-83962-7_18
