# Supplementary material for: Phytoplasma infection induces changes in vibrational signals of Cacopsylla pyri: sex-specific shifts in frequency, amplitude, and timing
Source: BMC Zool. 2026 May 30;11:19. doi: 10.1186/s40850-026-00270-6 (PMC13227762; doi:10.1186/s40850-026-00270-6)
Supplement: Supplementary file 2 — Supplementary Material 2 [file 40850_2026_270_MOESM2_ESM.docx]

A3

A binomial GLMM (logit link, random intercept: female ID) was fitted.
For treatment UU, interruption showed no significant effect (−2.40 ± 1.53 SE, z = −1.57, p = 0.12). A negative binomial GLMM (reference: UU) showed no treatment effect (Intercept UU: 2.90 ± 0.32 SE, p < 0.001; UI: −0.066 ± 0.52 SE, p = 0.90;
II: 0.79 ± 0.51 SE, p = 0.12). Estimates for UI and II were not reliably estimable due to large standard errors. GLM (reference: UU) indicated shorter latencies in II before correction (−1.39 ± 0.64 SE, p = 0.028), but no pairwise contrast remained significant after Holm correction.
