# Supplementary material for: Phytoplasma infection induces changes in vibrational signals of Cacopsylla pyri: sex-specific shifts in frequency, amplitude, and timing
Source: BMC Zool. 2026 May 30;11:19. doi: 10.1186/s40850-026-00270-6 (PMC13227762; doi:10.1186/s40850-026-00270-6)
Supplement: Supplementary file 3 — Supplementary Material 3 [file 40850_2026_270_MOESM3_ESM.docx]

Table 5. Descriptive statistics of vibrational signal parameters in infected and uninfected *Cacopsylla pyri* based on all individuals in the dataset. Values represent individual-level means per insect, summarised as mean ± SD, median, and number of individuals (N). Descriptive statistics are based on raw values aggregated at the individual level. Due to skewed distributions and unbalanced sampling across regions and individuals, descriptive measures may differ from model-based estimates.

| **Parameter** | **Sex** | **Infection** | **Median** | **Mean ± SD** | **N** |
| --- | --- | --- | --- | --- | --- |
| Chirp duration (s) | ♀ | infected | 0.0916 | 0.132 ± 0.027 | 10 |
| Chirp duration (s) | ♀ | uninfected | 0.130 | 0.199 ± 0.242 | 10 |
| Dominant frequency (kHz) | ♀ | infected | 3.223 | 4.882 ± 0.264 | 10 |
| Dominant frequency (kHz) | ♀ | uninfected | 5.518 | 4.528 ± 0.707 | 10 |
| Inter-chirp pause (s) | ♀ | infected | 0.936 | 0.952 ± 0.141 | 10 |
| Inter-chirp pause (s) | ♀ | uninfected | 0.766 | 0.725 ± 0.247 | 10 |
| Peak amplitude (kU) | ♀ | infected | 2.188 | 2.118 ± 0.551 | 10 |
| Peak amplitude (kU) | ♀ | uninfected | 1.925 | 1.749 ± 0.938 | 10 |
| Chirp duration (s) | ♂ | infected | 0.083 | 0.083 ± 0.015 | 10 |
| Chirp duration (s) | ♂ | uninfected | 0.079 | 0.081 ± 0.015 | 10 |
| Dominant frequency (kHz) | ♂ | infected | 2.922 | 3.005 ± 1.404 | 10 |
| Dominant frequency (kHz) | ♂ | uninfected | 2.635 | 2.823 ± 1.321 | 10 |
| Inter-chirp pause (s) | ♂ | infected | 0.573 | 1.765 ± 3.457 | 10 |
| Inter-chirp pause (s) | ♂ | uninfected | 0.542 | 1.110 ± 1.954 | 10 |
| Call (type ii) duration (s) | ♂ | infected | 22.926 | 23.461 ± 4.024 | 5 |
| Call (type ii) duration (s) | ♂ | uninfected | 22.208 | 22.235 ± 2.659 | 5 |
| Peak amplitude (kU) | ♂ | infected | 1.225 | 1.158 ± 0.450 | 10 |
| Peak amplitude (kU) | ♂ | uninfected | 1.308 | 1.350 ± 0.202 | 10 |
| Trill duration (s) | ♂ | infected | 16.962 | 16.127 ± 2.511 | 5 |
| Trill duration (s) | ♂ | uninfected | 15.678 | 15.540 ± 2.136 | 4 |
| Trill peak amplitude (kU) | ♂ | infected | 2.894 | 2.851 ± 1.048 | 5 |
| Trill peak amplitude (kU) | ♂ | uninfected | 3.148 | 3.206 ± 0.638 | 4 |
