# Supplementary material for: Phytoplasma infection induces changes in vibrational signals of Cacopsylla pyri: sex-specific shifts in frequency, amplitude, and timing
Source: BMC Zool. 2026 May 30;11:19. doi: 10.1186/s40850-026-00270-6 (PMC13227762; doi:10.1186/s40850-026-00270-6)
Supplement: Supplementary file 4 — Supplementary Material 4 [file 40850_2026_270_MOESM4_ESM.docx]

Table 4. Results of generalized linear mixed models testing the effect of ‘*Candidatus* Phytoplasma pyri’ infection on vibrational signal parameters of *Cacopsylla pyri* males and females. Gamma GLMMs with a log link were used for continuous positive response variables; individual ID was included as a random effect. Estimates are shown on the link scale. Biological effect directions are interpreted from the response-scale estimated marginal means in Tables 1 and 2. Significant effects (*p* < 0.05) are highlighted in bold.

| Signal type | Variable  (Response) | Sex | Estimate ± SE | z | p | Random  effect (ID) SD |
| --- | --- | --- | --- | --- | --- | --- |
| Call (type ii) | Duration (s) | ♂ | -0.050 ± 0.054 | 0.94 | 0.353 | 0.048 |
| Between Chirps | Pause (s) | ♀ | 0.47 ± 0.18 | 2.61 | **0.012** | 0.389 |
| Between Chirps | Pause (s) | ♂ | -0.698 ± 0.405 | 1.72 | 0.084 | 0.838 |
| Chirps | Peak amplitude (U) | ♀ | -0.287 ± 0.239 | 1.20 | 0.229 | 0.574 |
| Chirps | Peak amplitude (U) | ♂ | 0.41 ± 0.20 | 2.07 | **0.039** | 0.378 |
| Chirps | Dominant frequency (Hz) | ♀ | -0.162 ± 0.043 | -3.81 | **0.00014** | 0.482 |
| Chirps | Dominant frequency (Hz) | ♂ | 0.195 ± 0.226 | 0.87 | 0.387 | 0.482 |
| Trills | Peak amplitude (U) | ♂ | 0.196 ± 0.169 | 1.16 | 0.248 | 0.273 |
| Trills | Dominant frequency (Hz) | ♂ | 0.010 ± 0.227 | 0.04 | 0.966 | 0.481 |
